# Promoting Physical Activity in Older Adults With Type 2 Diabetes *via* an Anthropomorphic Conversational Agent: Development of an Evidence and Theory-Based Multi-Behavior Intervention

**DOI:** 10.3389/fpsyg.2022.883354

**Published:** 2022-07-12

**Authors:** Nuno Pimenta, Isa Brito Félix, Diogo Monteiro, Marta Moreira Marques, Mara Pereira Guerreiro

**Affiliations:** ^1^Sport Sciences School of Rio Maior, Polytechnic Institute of Santarém, Santarém, Portugal; ^2^Interdisciplinary Centre for the Study of Human Performance, Faculty of Human Kinetics, Cruz-Quebrada, Portugal; ^3^Centro de Investigação Interdisciplinar em Saúde, Universidade Católica Portuguesa, Lisbon, Portugal; ^4^Nursing Research, Innovation and Development Centre of Lisbon (CIDNUR), Nursing School of Lisbon, Lisbon, Portugal; ^5^ESECS – Polytechnic of Leiria, Leiria, Portugal; ^6^Research Centre in Sport, Health and Human Development (CIDESD), Vila Real, Portugal; ^7^Life Quality Research Centre (CIEQV), Leiria, Portugal; ^8^Comprehensive Health Research Centre (CHRC), NOVA Medical School, Universidade Nova de Lisboa, Lisbon, Portugal; ^9^Centro de Investigação Interdisciplinar Egas Moniz (CiiEM), Instituto Universitário Egas Moniz, Monte de Caparica, Portugal

**Keywords:** conversational agent, older adults, type 2 diabetes, physical activity, intervention development, behavior change techniques, self-determination theory

## Abstract

**Introduction:**

Anthropomorphic conversational agents (ACA) are a promising digital tool to support self-management of type 2 diabetes (T2D), albeit little explored. There is a dearth of literature on the detailed content of these interventions, which may limit effectiveness and replication. Our aim is to describe the development of an evidence and theory-based intervention to improve physical activity in older adults with T2D, subsumed in a multi-behavior intervention *via* a mobile application with an ACA.

**Methods:**

Overall decisions on the multi-behavior intervention design, such as the use of standardized behavior change techniques (BCTTv1), guided the development of the physical activity component. Firstly, recommendations on ambulatory activity were used to select the target behavior (walking). Meta-research on effective behavior change techniques (BCTs) was then identified. One meta-analysis linked effective BCTs with the three basic psychological needs of the self-determination theory (SDT). This meta-analysis, taken together with additional evidence on SDT, led to the selection of this theory to inform the design. BCTs were extracted from meta-research; we selected the most appropriate to be operationalized *via* the conversational agent through multidisciplinary discussions. Rules governing the dialogue flow and BCTs tailoring, taking the form “if some conditions hold then execute some action,” were derived based on the Basic Psychological in Exercise Scale (competence, autonomy, and relatedness scores), in conjunction with published evidence and multidisciplinary discussions.

**Results:**

Thirteen BCTs were implemented in the prototype *via* the ACA (e.g., goal setting behavior 1.1). Six if-then rules were derived and depicted in the dialogue steps through process flow diagrams, which map how the system functions. An example of a rule is “If competence score ≤ 10 then, apply BCT 1.1 with 500 steps increments as options for the daily walking goal; If competence score > 10 then, apply BCT 1.1 with 1,000 steps increments as options for the daily walking goal.”

**Conclusion:**

Evidence and SDT were translated into a mobile application prototype using an ACA to promote physical activity in older adults with T2D. This approach, which includes 13 BCTs and six if-then rules for their tailoring, may leverage the efforts of others in developing similar interventions.

## Introduction

The sustainability and quality of healthcare provision in many countries is threatened by a constellation of factors, such as aging, the rising burden of non-communicable diseases and shortage of health professionals. It is estimated that the number of older people in the European Union (EU) will increase significantly, from 90.5 million at the start of 2019 to 129.8 million by 2050 (European Union, 2020). Public expenditure on health and long-term care has been increasing over the last decades in all EU Member States (European Union, 2020). These factors have driven the reengineering of health care delivery and the role of digital health technology.

Diabetes is one of the fastest growing health issues. Globally it affects around 537 million adults; type 2 diabetes (T2D) accounts for over 90% of all diabetes cases and has an increasing prevalence by age (International Diabetes Federation, 2021). Cardiovascular diseases are the leading cause of morbidity and mortality for individuals with diabetes accounting for an estimated cardiovascular-related cost of $37.3 billion per year, associated with diabetes (American Diabetes Association, 2018).

Furthermore, additional common metabolic conditions often coexist with T2D (e.g., hypertension and dyslipidemia) confers an increased health risk in this specific group (American Diabetes Association, 2021a). Diabetes-related health expenditures, irrespective of being borne by people living with diabetes, their families, or the health system, grew globally from USD 232 billion in 2007 to USD 966 billion in 2021 for adults aged 20–79 years, representing a 316% increase over 15 years (International Diabetes Federation, 2021).

Sustained hyperglycemia in persons with T2D also increases the risk of other complications, such as renal failure and retinopathy (International Diabetes Federation, 2021). T2D may also engender distress, understood as “negative emotional or affective experience resulting from the challenge of living with the demands of diabetes” (Skinner et al., 2020), and impaired health-related quality of life (Cannon et al., 2018).

It has been estimated that persons with diabetes spend fewer than 6 h per year consulting with healthcare professionals (Holt and Speight, 2017), which illustrates the importance of empowering and supporting these persons to actively self-manage the condition. Physical activity and other lifestyle behaviors fall under the remit of self-management, as they are dependent on the daily role taken by persons living with diabetes (American Diabetes Association, 2021b). Such behaviors, as regular physical activity and healthy eating are the cornerstone of T2D management (American Diabetes Association, 2021b; Kanaley et al., 2022).

Physical activity and exercise have been endorsed as a treatment or adjunct therapy for T2D and, at least, 25 other health conditions, including some T2D cardiometabolic comorbidities (Pedersen and Saltin, 2015; Shah et al., 2021). Sound evidence-based recommendations for physical activity and exercise for persons with T2D have been released (American Diabetes Association, 2021b; Kanaley et al., 2022). Simple physical activity behaviors such as walking one mile per day, or more (≥1.6 km/day), may provide a two-fold reduction in adjusted risk of all-cause mortality and a five-fold reduction in adjusted risk of non-coronary cardiovascular disease (CVD) death in older adults with diabetes (Smith et al., 2007).

Digital Behavior Change Interventions are a coordinated sets of activities or products designed to change specified behavioral patterns (e.g., physical activity) of individuals through digital technology, such as mobile applications, wearable technology (e.g., activity trackers), or websites. Digital Behavior Change Interventions are a promising approach for empowering diabetes self-management, as they have the capability to deliver personalized solutions to influence complex and challenging health behaviors (Michie et al., 2017). These digital interventions can support persons with diabetes to engage in physical activity, healthy eating, and overall disease management behaviors, intended to improve health outcomes and reduce complications (Fleming et al., 2020). Although T2D has a growing prevalence in older people, it has been recognized that mobile applications have limited usability for this group, which may hinder their use (Arnhold et al., 2014; Berenguer et al., 2017).

A meta-analysis of mobile applications for T2D, included 6 randomized controlled trials, with a total of 1,022 participants, and found an overall efficacy in reducing glycated hemoglobin (HbA1c), with a mean 0.40% decrease (95% CI 0.11 to 0.69%; Cui et al., 2016). Typically, a change in glycated hemoglobin of 0.5% is considered clinically significant (Little and Rohlfing, 2013). As for the cost-effectiveness of T2D digital interventions, a systematic review, included seven full economic evaluations, three of which comprised self-management support, albeit non-automated. Of these, the two studies that reported cost per quality-adjusted life-year (QALY) gained were cost-effective (Rinaldi et al., 2020). Although economic evaluation of these interventions is still in a nascent stage, it shows encouraging results, favoring resource allocation to digital diabetes self-management interventions.

Conversational agents, defined as computer programmes designed to simulate two-way human conversation using language (speech and/or text), potentially supplemented with non-language modalities are regarded as a promising approach to support diabetes self-management (Guerreiro et al., 2021). They may, for example, be more user friendly for people with lower literacy. These agents can be integrated in multiple devices, including mobile phones. Virtual human is another term to describe anthropomorphic conversational agents. A meta-analysis demonstrated the effectiveness of virtual humans in patient-facing systems, based on 26 controlled studies. Future accumulation of research may help to overcome the moderate heterogeneity in study results (Chattopadhyay et al., 2020). Notably, no trial involving long-term self-management support in T2D was included in this meta-analysis.

More recently, Luo et al. reported a dearth of research on conversational agents targeting physical activity in persons with T2D. In particular, published interventions do not always rely on behavior change theory (Luo et al., 2021), which may curtail their effectiveness, nor explicitly present their active components, which limits replication and knowledge transfer.

One example of the use of conversational agents in T2D management is the VASelfCare project (2018/01-2020/03).^1^ This project developed a multi-behavior change digital intervention for older people with T2D, *via* an anthropomorphic conversational agent and a connected web-based dashboard for health professionals. Vitoria, a 3D female virtual human, was designed as a coach for three target behaviors: medication taking, physical activity, and healthy eating, resorting to design principles for older adults. The overall intervention has been described previously (Balsa et al., 2020; Guerreiro et al., 2020) and the development of the medication taking component detailed elsewhere (Félix et al., 2019). The current paper focuses on the development of the physical activity component, a key behavior in diabetes management in older adults (Bellary et al., 2021).

The Medical Research Council (MRC) framework (Skivington et al., 2021) for developing and evaluating complex intervention, which guided the VASelfCare project, recommends an accurate process of development drawing on existing theories, modeling of process and outcomes, followed by feasibility assessment.

The importance of theory-based interventions for promoting physical activity in persons with T2D has been recently underscored (Konerding and Szel, 2021). Theory-based interventions are recommended as it provides assumptions about why interventions differently affect health behavior (Gourlan et al., 2016). The effectiveness of the intervention depends on the theory selected and how design and implementation of the intervention fits to the theoretical constructs. Additionally, only a small proportion of interventions publish the link between theory and behavior change techniques (BCTs), despite the recommendations to improve the transparency of theory-based interventions (Michie and Abraham, 2004; Abraham et al., 2014; National Institute for Health and Care Excellence, 2014). To enable interventions to be evaluated and implemented, BCTs should be well specified, increasing their accurate replication.

Enhancing the importance of adequate description of the interventions, the MRC endorses the publication of the intervention development process as it allows others to establish links between this process and the subsequent success of interventions and learn about the endeavors of this approach, which may be useful for developers (O’Cathain et al., 2019). Currently, there are limited examples in the literature of detailed descriptions of how systematic processes of developing digital health behaviors change interventions (Encantado et al., 2021), including in physical activity.

This paper describes the development of an evidence and theory-based intervention to improve physical activity in older adults with T2D, subsumed in a multi-behavior intervention *via* a mobile application with an anthropomorphic conversational agent.

## Materials and Methods

The design of the VASelfCare intervention is described below, focusing firstly on the overall design, and then on the procedures to design the physical activity component, as part of the multi-behavior intervention (medication-taking, physical activity and healthy eating). The overall design of the intervention framed the development of the physical activity component and is therefore critical to its understanding. These procedures for the latter detail the use of theory and evidence for deriving the content of the physical activity component and rules to tailor them.

### Overall Design of the Multi-Behavior Intervention

The anthropomorphic conversational agent, Vitoria, developed within the VASelfCare project, was designed to support behavior change through daily interactions with users. Based on the literature (e.g., Bickmore et al., 2010), users are offered the possibility of interacting with Vitoria once a day only. Vitória is capable of speaking European Portuguese and expressing emotions through facial and body animations; verbal content is supplemented with subtitles to help reduce potential communication barriers such as hearing deficits. User’s input consists of a set of options depicted in response buttons or through values recorded. For details on the IT development of the anthropomorphic conversational agent prototype refer to Balsa et al. (2020) and Guerreiro et al. (2020).

An overall design choice, regardless of the component, was to address each target behavior in two stages: in the evaluation stage Vitoria collects data to tailor the intervention content in the subsequent follow-up stage, which purports to promote or maintain the behaviors.

Tailoring is employed in this paper as “any combination of information and behavior change strategies intended to reach one specific person based on characteristics that are unique to that person related to the outcome of interest and derived from an individual assessment” (Kreuter et al., 2000). Across the three components of the VASelfCare intervention, tailoring relies not only on data from the evaluation stage, but also from previous interactions, and addresses both general information about diabetes, to improve health literacy, and the selection of BCTs, as explained later in this section.

**Figure 1:**
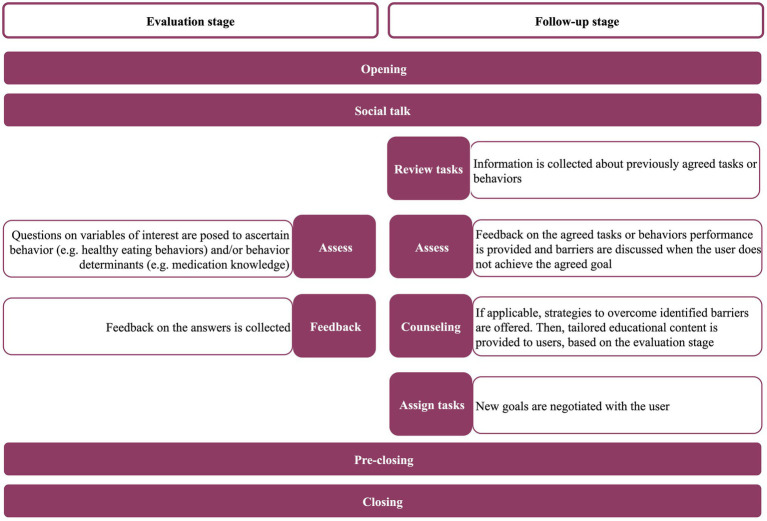
Steps of each daily interaction in the evaluation and follow-up stages across the three components (medication taking, physical activity and healthy eating).

Each daily interaction was structured in sequential steps based on the literature (Bickmore et al., 2005). In the evaluation stage, the sequential steps are depicted in Figure 1 (opening, social talk, assess, feedback, pre-closing and closing). The “opening” and “social talk” steps involve greeting the user and inquiries about the general emotional and physical state, respectively. Finally, the content of the next interaction is described in the “pre-closing” step and a farewell is delivered (“closing”). The dialogue in the follow-up stage has three additional steps, “review tasks,” “counseling,” and “assign tasks,” also described in Figure 1.

**Figure 2:**
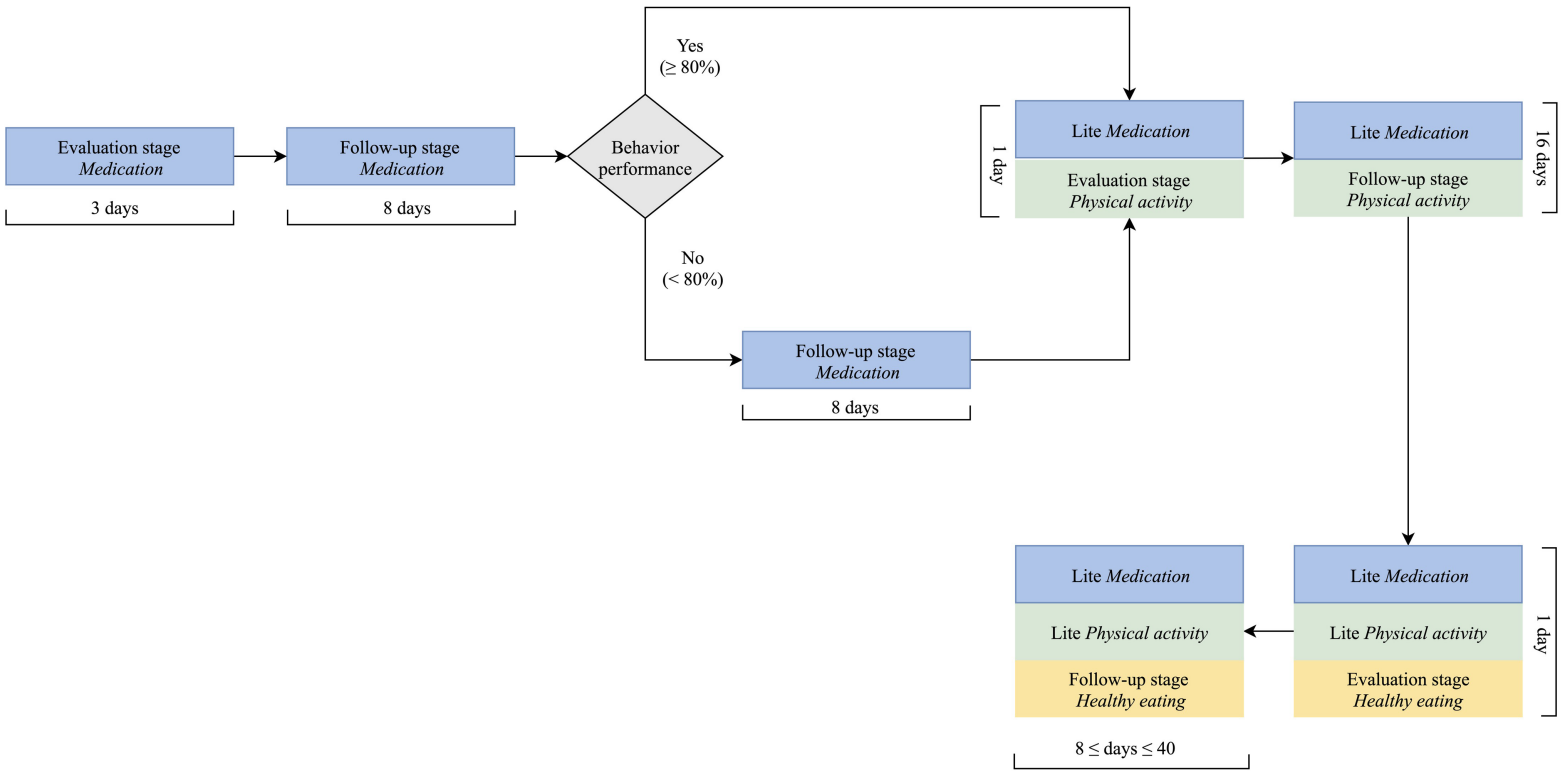
Multi-behavior intervention design (assumption: one interaction per day).

A critical design decision was how to combine the physical activity component with the remaining components of the intervention: medication taking and healthy eating. There is little guidance on the best approach to design digital multi-behavior interventions; general recommendations indicate that interventions targeting behaviors requiring inaction (such as not eating high fat food) and those requiring action (such as increasing physical activity or taking medication) should not be pursued concomitantly (Albarracín et al., 2018). From a practical standpoint, pursuing three behaviors at the same time from the outset of the intervention would increase the length of each interaction to a point that was deemed detrimental for engagement. Taken together, these two aspects determined a stepwise approach, in which medication taking is firstly addressed, then the physical activity component added, while reducing the intensity of the medication taking component; as depicted in Figure 2, the same approach was employed for the healthy eating component. A less intensive intervention in each component, designated as “lite” (Figure 2), comes up after firstly, addressing each target behavior more intensively. “Lite” interventions have reduced number of dialogue steps and provide feedback to users based on weekly data (e.g., average medication taken in the last week). They encompass repeated collection of evaluation data, such as on medication knowledge and healthy eating behaviors, to enable intervention tailoring in the long term.

### Design of the Physical Activity Component as Part of the Multi-Behavior Intervention

For the physical activity component, the VASelfCare intervention focused on ambulatory activity, mostly walking, as it has been endorsed for persons with T2D (Moghetti et al., 2020). This target group is regarded at high risk for exercise-related complications (Burr et al., 2012). Hence, we refrained from including recommendations of moderate to vigorous exercise, as the anthropomorphic conversational agent is unable to assess exercise tolerance and monitor exercise. Walking or walking-related activities are part of daily routine and considered feasible and low risk for older persons with T2D (Dasgupta et al., 2017; Barbosa et al., 2020). Correspondingly, daily step counting was chosen as a primary marker of this lower-level target behavior, focusing on helping users to achieve healthy levels of ambulatory physical activity (Tudor-Locke et al., 2011b).

An increase of 4,000 steps/day on a 5-day average step counts, from baseline, was found to be the threshold to elicit a clinically meaningful reduction on glycated hemoglobin in persons with type 2 diabetes, in a 24-week pedometer-based physical activity intervention (Van Dyck et al., 2013). More recently, a randomized controlled trial, resorting to a simple physician-delivered step count prescription strategy incorporated into routine clinical practice, showed that an average increase of 1,220 steps/day elicited a significant increase in insulin sensitivity and reduction of glycated hemoglobin (Dasgupta et al., 2017). A systematic review of studies in adults from non-clinical populations found that an increase of 1,000 steps/day elicited a reduction in the risk of all-cause mortality and cardiovascular morbidity and mortality (Hall et al., 2020). Data are currently lacking to identify an optimal health enhancing minimum threshold of daily step counts, still, health benefits can be found below 10.000 steps/day (Hall et al., 2020).

A walking cadence of 100 steps/min has been considered to be a reasonable empirical value for adults, indicative of walking at approximately the lower limit of the moderate-intensity spectrum (i.e., ≈ 3 METs; Tudor-Locke et al., 2011b; Tudor-Locke and Rowe, 2012). A target step counts of 7,000 steps/day, considering a step cadence of about 100 steps/min, is equivalent to approximately 70 min of walking per day. However, not all daily steps are expected to be performed at a cadence of 100 steps/min and, therefore, within the spectrum of moderate-intensity physical activity (Tudor-Locke et al., 2011a). Still, the goal of 7,000 steps/day has been shown to be consistent with physical activity guidelines for adults (Tudor-Locke et al., 2011c), particularly with the recommendation of 150 to 300 min/week of moderate–intensity, which has been recently endorsed also for persons with T2D (Kanaley et al., 2022), and has already been used and found beneficial in interventions in older and clinical populations (Gardner et al., 2021; Saad et al., 2021), including in persons with T2D (Rossen et al., 2021).

According to the rationale presented and the selected physical activity primary surrogate, a pedometer (New-Lifestyles NL-2000i Activity Monitor; New-Lifestyles Inc., Euless, TX, United States) was chosen to count daily steps. This device provides a reliable measure of this marker (Crouter et al., 2005; Grant et al., 2008; Tedesco et al., 2019); additionally, self-monitoring *via* a pedometer has been found useful on its own to increase daily step counts (Bravata et al., 2007), and beneficial for persons with T2D (Idowu et al., 2021). To foster scalability (future use across a range of devices), daily steps are inputted directly by users in the application interface.

A requirement for the content of the intervention was using behavior change techniques (BCTs) from an established taxonomy across the three target behaviors addressed by the intervention. A BCT is “an observable, replicable and irreducible component of an intervention designed to alter or redirect causal processes that regulate behavior (Michie et al., 2013). The Behaviour Change Taxonomy version 1 (BCTTv.1) was selected to specify these active ingredients of the intervention. BCTTv1 is an extensive hierarchically ordered and reliable taxonomy of 93 distinct BCTs that are categorized into 16 groups. For the physical activity component, the literature offered meta-research on effective BCTs (Cradock et al., 2017; Gillison et al., 2019), and therefore selecting BCTs using a systematic and structured process, as described for the medication adherence component (Félix et al., 2019), was deemed unnecessary.

In particular, one of the meta-analysis linked BCTs from the BCTTv.1 with the three basic psychological needs encompassed in the self-determination theory (Gillison et al., 2019).

Self-determination Theory (SDT) is a broad meta-theory of motivation. Central to the theory is the distinction between autonomous (self-determined) and controlled (non-self-determined) forms of motivation, which are further specified in a motivational continuum ranging from the most autonomous form of motivation (intrinsic motivation) to the most controlled form of motivation (external motivation; Ryan and Deci, 2017).

Autonomous motivation refers to a motivation that is based on self-endorsed reasons to choose and pursue a goal or action, when one has a full sense of willingness, volition, and choice, independently of the activity. One can feel autonomously motivated when doing an activity that is intrinsically enjoyable or fun (intrinsic motivation) or because that activity or goal genuinely fits their sense of self and values (integrated motivation). When acting with autonomy, a person is fully functioning, willingly engaged in activity with awareness and congruence, and able to harness vitality in the self-regulation of action (Ryan and Deci, 2017). In contrast, controlled motivation refers to reasons for acting that are not self-endorsed, that are subject to some form of pressure, either external by others or internally by the individual (e.g., feelings of guilt, for an external reward). Research in diverse life domains suggests that more autonomous, relative to controlled, motives are not only associated with, but essential to, a variety of positive outcomes (Ryan and Deci, 2017).

Additionally, the authors argue that all human beings have three basic psychological needs - Autonomy, Relatedness, Competence - that need to be satisfied for one to feel autonomously motivated and, consequently, to achieve optimal performance, psychological health and well-being (Ryan and Deci, 2017; Vansteenkiste et al., 2020). The need for competence reflects the need to feel effectance and mastery over tasks and behavior; the need of autonomy reflects the need to feel a sense of ownership and choice in acting; lastly, the need of unconditional support and connectedness with others is reflected by relatedness. It is posited that when the three basic psychological needs are satisfied, autonomous motivation and mental health are enhanced (Ryan and Deci, 2000).

There is strong evidence on the effectiveness of interventions based on the self-determination theory across a wide range of health domains, including physical activity (Teixeira et al., 2012). Primary research also supports the use of this theory as successful in increasing physical activity in older adults with type 2 diabetes (Koponen et al., 2018). Taken together, this evidence led to choosing the self-determination theory (SDT) to inform the design of the physical activity component. A more recent meta-analysis corroborates this choice; Ntoumanis et al. (2021) showed that SDT-based interventions (*n* = 73) positively affect health behaviors at the end of the intervention period and at the follow-up, in particular physical activity.

BCTs were extracted and listed from meta-research (Cradock et al., 2017; Gillison et al., 2019). As explained, Gillison et al. (2019) presented BCTs to promote psychological needs, satisfaction and motivation in health interventions based on the self-determination theory; Cradock et al. (2017) identified the BCTs associated with changes in glycated hemoglobin and body weight in persons with T2D.

From the listed BCTs, we selected the most appropriate to be operationalized *via* the conversational agent through multidisciplinary discussion, inspired by the practicality criterion, as defined by Michie et al. (2014) - extent to which the intervention can be delivered as designed through the means intended to the target population. In essence, foci of discussions were whether it was practicable to deliver the listed BCTs through the conversational agent. The team included expertise from the disciplines of sport sciences, psychology, nursing, pharmacy, and informatics.

Vitoria dialogues resort to an artificial intelligence rule-based engine. Rules derived to control the dialogue flow take the form “if some conditions hold then execute some action,” where the conditions may include context information regarding the interaction (e.g., user characteristics or the date when interaction takes place) and the action represents the subsequent act to be performed by the conversational agent. These rules were informed by the Basic Psychological in Exercise Scale (Moutão et al., 2012), published evidence (BCTs emanating from meta-research, as explained) and multidisciplinary discussions. The Basic Psychological in Exercise Scale is used to assess psychological needs for exercise underlying Self-Determination Theory (Ryan and Deci, 2017); it comprises a total of 12 items grouped into three factors: autonomy (four items), competence (four items) and relatedness (four items). Responses are provided on a 5-point Likert-type scale ranging from 1 (strongly disagree) to 5 (strongly agree). The maximum score for each construct is 20.

## Results

### Selection and Operationalization of BCTs in the Physical Activity Component

Thirteen BCTs were selected for implementation in the mobile application prototype *via* the conversational agent. Table 1 details these BCTs and their operationalization according to the interaction steps in the follow-up stage. In the evaluation stage, BCTs are not applicable as its purpose is not changing behavior.

**Table 1:** Description of the BCTs and operationalization used by the anthropomorphic conversational agent.

**Behavior change techniques and definition (BCTTv.1)**	**Operationalization**	**Dialogue step**
**Goal setting (behavior**; **1.1)** *Set or agree on a goal defined in terms of the behavior to be achieved*	Vitoria collaboratively defines with the user the number of daily steps to be achieved	Assign tasks
**Problem-solving (1.2)** *Analyze, or prompt the person to analyze, factors influencing the behavior and generate or select strategies that include* *overcoming barriers and/or increasing facilitators*	Vitoria lists the potential barriers to walking as agreed and, based on the selected factors influencing the behavior, offers options to overcome barriers or enhance facilitators	Counseling
**Review behavior goal(s**) (**1.5)** *Review behavior goal(s) jointly with the person and consider modifying goal(s) or behavior change strategy considering achievement. This may lead to re-setting the same goal, a small change in that goal or setting a new goal instead of (or in addition to) the first, or no change*	When the goal (i.e., number of steps) is not achieved, Vitoria reviews it collaboratively with the user to define a new goal (i.e., number of steps) or keeping the same goal	Assign tasks
**Feedback on behavior (2.2)** *Monitor and provide informative or* *evaluative feedback on performance of* *the behavior (*e.g.*, form, frequency, duration, intensity)*	Vitoria provides verbal and visual information on daily step counts, using a helpful-cooperative communication style and *via* a chart	Assess
**Self-monitoring of behavior (2.3)** *Establish a method for the person to* *monitor and record their behavior(s) as* *part of a behavior change strategy*	Vitoria asks the user to input step counts, measured by a pedometer	Review tasks
**Social support (unspecified**; **3.1)** *Advise on, arrange or provide social support (*e.g.*, from friends, relatives, colleagues,’ buddies’ or staff) or non-contingent praise or reward for performance of the behavior. It includes encouragement and counseling, but only when it is directed at the behavior*	Vitoria advises the user to invite friends or family members to go for a walk	Counseling
**Instruction on how to perform the behavior (4.1)** *Advise or agree on how to perform the behavior (includes ‘Skills training’)*	Vitoria advises on how to accommodate physical activity in the daily routine, such as walking the dog, exercise while watching TV and parking further away from the destination	Counseling
**Information about health consequences (5.1)** *Provide information (*e.g.*, written, verbal, visual) about health consequences of performing the behavior*	Vitoria highlights the positive consequences of walking and the negative consequences of sedentarism	Counseling
**Information about social and environmental consequences (5.3)** *Provide information (*e.g.*, written, verbal, visual) about social and environmental consequences of performing the behavior*	Vitoria highlights that walking is considered important to people’s health and for the sustainability of the planet	Counseling
**Information about emotional consequences (5.6)** *Provide information (*e.g.*, written, verbal,* *visual) about emotional consequences of* *performing the behavior*	Vitoria focuses on the psychological benefits of physical activity (e.g., well-being)	Counseling
**Restructuring the physical environment (12.1)** *Change, or advise to change the physical* *environment to facilitate* *performance of the wanted behavior or* *create barriers to the unwanted behavior* *(other than prompts/cues, rewards and* *punishments)*	Vitoria advises the user to leave the walking shoes or walking aids at sight (e.g., by the entrance door instead of locked in a closet)	Counseling
**Restructuring the social environment (12.2)** *Change, or advise to change the social environment to facilitate performance of the wanted behavior or create barriers to the unwanted behavior (other than prompts/cues, rewards, and punishments)*	Vitoria advises the user to persuade family or friends to accompany him or her in walks	Counseling
**Verbal persuasion about capability (15.1)** *Tell the person that they can successfully perform the wanted behavior, arguing against self-doubts, and asserting that they can and will succeed*	Vitoria asserts that the user can increase step counts despite potential difficulties or limitations	Assign tasks

The number between brackets refers to the BCTTv1.

### Rules for Tailoring BCTs in the Physical Activity Component

This section presents rules for tailoring BCTs in the physical activity component. Firstly, the “if-then” rules are summarized in Table 2, then rules are put into context in the interaction steps through process flow diagrams, which map how the system functions.

**Table 2:** Decision rules for tailoring BCTs.

Example	Interaction step	Rule	Related BCTs
A	Day 1, Assign tasks (see Figure 3)	If competence score ≤ 10 Then, apply BCT 1.1 with 500 steps increments as options for setting the daily walking goal	Goal setting behavior (1.1)
		If competence score > 10 Then, apply BCT 1.1 with 1,000 steps increments as options for setting the daily walking goal	Goal setting behavior (1.1)
B	Day 2 and subsequent even days, Counseling (see Figure 4)	If competence score < (autonomy score AND relatedness score) Then, BCTs 4.1, 12.1 AND 15.1 applied during 8 days	Instruction on how to perform a behavior (4.1) Restructuring the physical environment (12.1) Verbal persuasion about capability (15.1)
		If autonomy score < (competence score AND relatedness score) Then, BCTs 5.1, AND 5.3 applied during 5 days	Information about health consequences (5.1) Information about social and environmental consequences (5.3)
		If relatedness score < (competence score AND autonomy score) Then, BCT 3.1, applied during 2 days	Social support (unspecified; 3.1)
C	Day 3 and subsequent odd days, Assess/Counseling (see Figure 5)	If behavior goal achieved Then, apply BCTs corresponding to the construct with the lowest score (competence OR autonomy OR relatedness)	See example B
		If behavior goal not achieved Then, apply BCT 1.2 AND (BCT 3.1 OR 4.1 OR 5.1 OR 5.3 OR 5.6 OR 12.1 OR 12.2)	Problem-solving (1.2) Social support (unspecified; 3.1) Instruction on how to perform a behavior (4.1) Information about health consequences (5.1) Information about social and environmental consequences (5.3) Information about emotional consequences (5.6) Restructuring the physical environment (12.1) Restructuring the social environment (12.2)
D	Day 3 and subsequent odd days, Counseling/Assign tasks (see Figure 5)	If Δ ≥ ± 2000 steps in relation to the agreed goal Then, apply BCT 1.5 (based on step counts achieved on the previous day OR step counts of the agreed goal two days before) using increments determined by the competence score	Review behavior goal(s) (1.5)
		If Δ < ± 2000 steps in relation to the agreed goal Then, apply BCT 1.5 (based on the step counts observed in the previous day) using increments determined by the competence score	Review behavior goal(s) (1.5)
E	“Lite” version, Assess/Counseling (see Figure 8)	If average weekly goal not achieved Then, apply BCT 1.2 AND (BCT 3.1 OR 4.1 OR 5.1 OR 5.3 OR 5.6 OR 12.1 OR 12.2)	Problem-solving (1.2) Social support (unspecified; 3.1) Instruction on how to perform a behavior (4.1) Information about health consequences (5.1) Information about social and environmental consequences (5.3) Information about emotional consequences (5.6) Restructuring the physical environment (12.1) Restructuring the social environment (12.2)
F	“Lite” version, Counseling/Assign tasks (see Figure 8)	If Δ ≥ ± 2000 steps in relation to the average weekly goal Then, apply BCT 1.5 (based on average step counts of the last seven days OR step counts of the average weekly goal) using increments determined by the competence score	Review behavior goal(s) (1.5)
		If Δ < ± 2000 steps in relation to the average weekly goal Then, apply BCT 1.5 (based on the average step counts of the last seven days) using increments determined by the competence score	Review behavior goal(s) (1.5)

BCTs were operationalized differently on the first day of follow-up and on the subsequent even and odd days. Underlying this approach was the fact that the walking goal defined with Vitoria on any given day (D) pertains to the day after the interaction (D + 1); in other words, the walking goal entails the full steps of D + 1. Since the user can interact with Vitoria at any time on D + 1, goal achievement could be unduly compromised. Therefore, the goal set on day 1 was designed to be assessed on day 3 (Feedback on behavior, 2.2), by reporting the step counts on day 2, corresponding to a full day, and so forth.

#### Day 1 of Physical Activity Component: Follow-Up Stage

**Figure 3:**
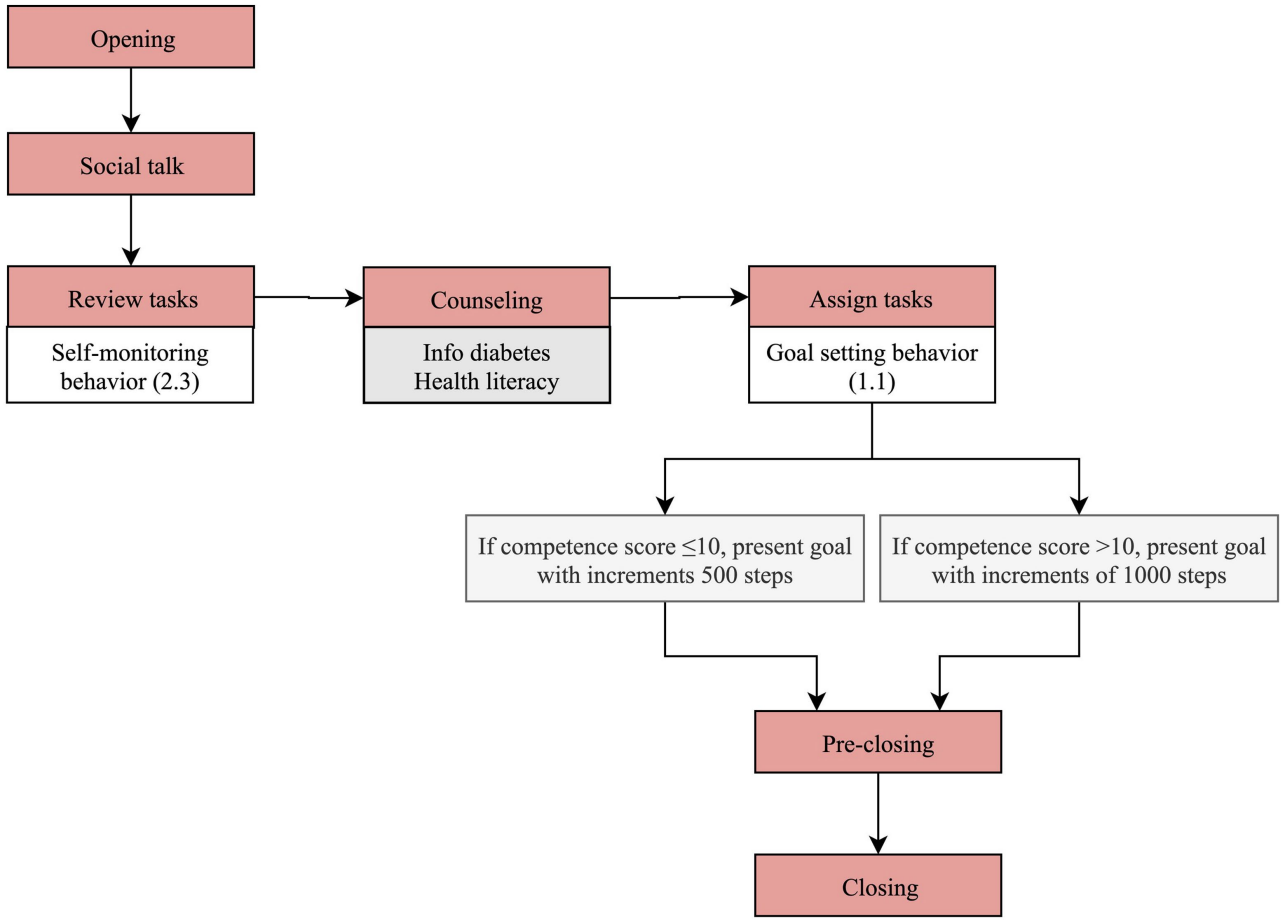
Dialogue flow in day 1 of physical activity component of the follow-up stage.

Figure 3 details the dialogue flow on day 1 of the follow-up stage according to the rules described above.

The first and last two steps (“opening” and “social talk” plus “pre-closing” and “closing,” respectively) are common in terms of content across the interactions, regardless of the day, as explained in the Materials and Methods section.

On the first day of follow-up, information is collected about the average step counts of the last 7 days, in “review tasks” (self-monitoring of behavior 2.3). At this stage no behavior goal has been set up yet, and therefore the “assess” step, in which feedback would be given, is skipped.

In the “counseling” step, Vitoria gives general information about T2D and complications, such as details on hypoglycemia symptoms and how to manage them, to improve health literacy.

Next, in the “assign tasks” step, Vitoria collaboratively agrees with the user on a goal for step counts for the next day (goal setting behavior 1.1). The three options presented are informed by the competence score, yielded in the evaluation phase, and the average step counts of the last 7 days, as shown in example A of Table 2. For example, if the user reports an average of 7,000 steps in the last 7 days and the competence score is >10, Vitoria suggests three goals for the next day: 8000, 7,000 and 6,000 steps.

#### Day 2 and Subsequent Even Days of the Physical Activity Component: Follow-Up Stage

**Figure 4:**
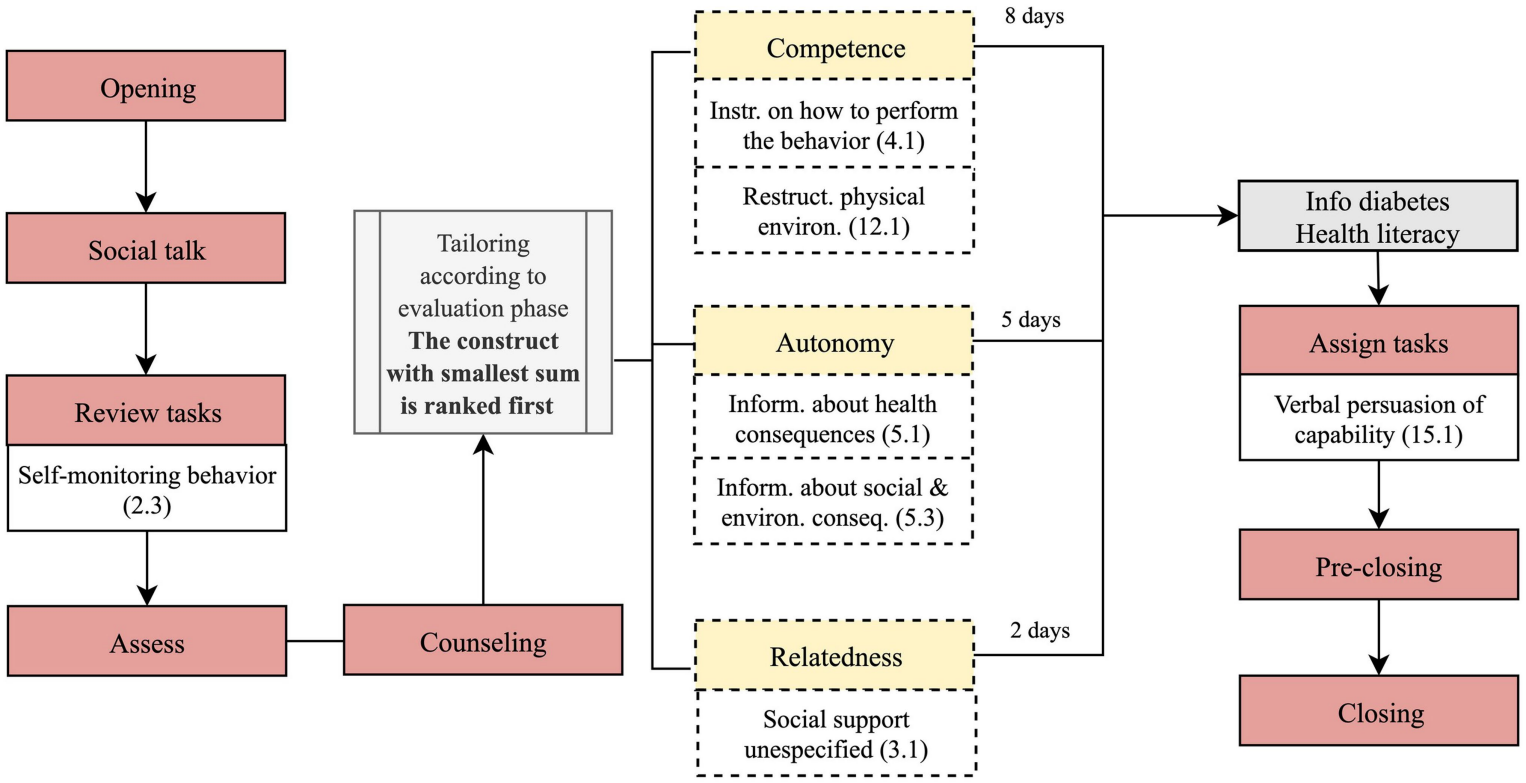
Dialogue flow on day 2 and even days of physical activity component of the follow-up stage.

On the second day and even days of the follow-up stage, Vitoria dialogue follows the steps detailed in Figure 4.

In the “review tasks” step, Vitoria asks the user to input the step counts recorded by the pedometer on the previous day (self-monitoring of behavior 2.3). This step is followed by the “assess” step, in which Vitoria reminds the user of the agreed goal (first day and odd days).

Then, information to improve competence, autonomy and relatedness is delivered stepwise throughout the intervention (“counseling” step), starting with the construct with the lowest score. For instance, in a user with a competence, autonomy and relatedness scores of 4, 12, 18, respectively, Vitoria will firstly address competence, encompassing a set of BCTs, as illustrated by example B, Table 2. The number of days allocated to each construct varies between two for relatedness and eight for competence, assuming one interaction per day, as already explained in section 2.1. Each construct is targeted in consecutive days, or interspersed with eliciting behavioral barriers, if the walking goal is not met, as assessed on odd days. When competence is addressed, Vitoria explains, for instance, how to use resources to walk, such as a walker, a cane, or a trekking pole (instruction on how to perform the behavior 4.1). Addressing autonomy entails, for example, information about the positive consequences of walking (information about health consequences 5.1). To promote relatedness, Vitoria suggests inviting a friend or a family member to walk or joining group classes (social support unspecified 3.1).

#### Day 3 and Subsequent Odd Days of the Physical Activity Component: Follow-Up Stage

**Figure 5:**
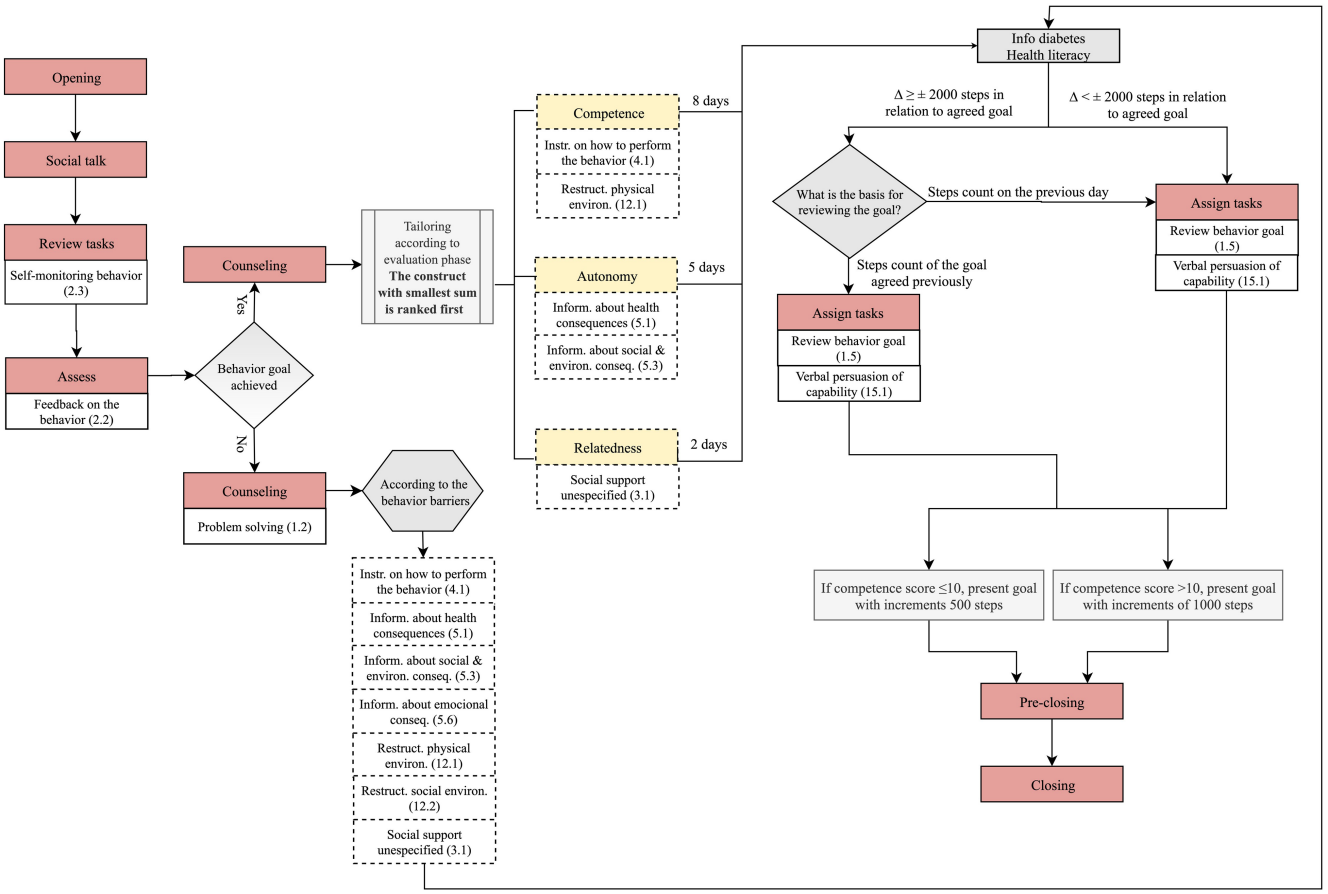
Dialogue flow in day 3 and odd days of physical activity component of the follow-up stage.

**Figure 6:**
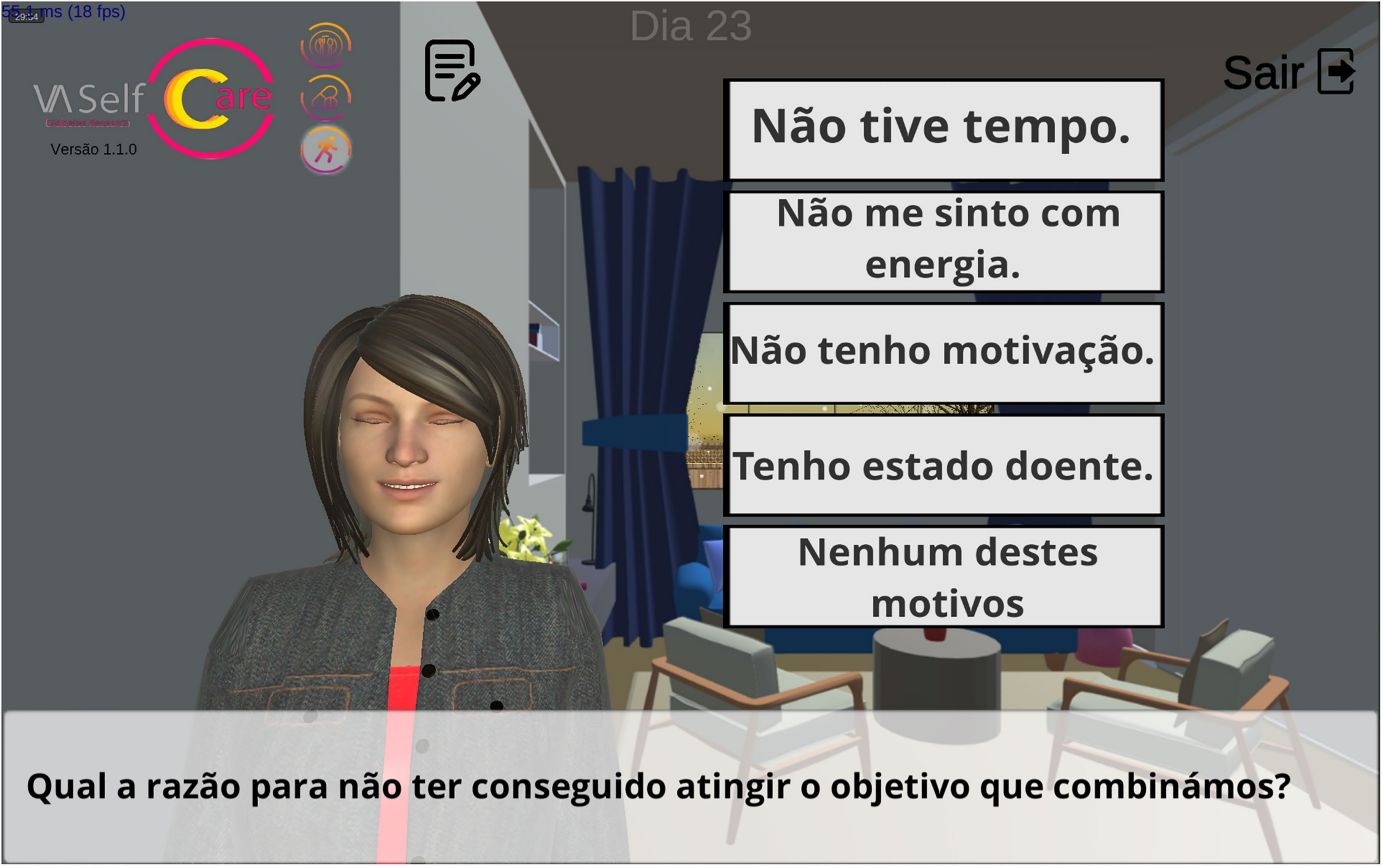
Interface of the mobile application: Vitoria addressing barriers to walking (problem-solving 1.2). Translation to English: What is the reason for not having achieved the goal we agreed upon? (subtitle); (A) I did not have time; (B) I do not feel energetic; (C) I am not motivated; (D) I have been sick and (E) None of these reasons (response options).

Figure 5 presents the dialogue flow on the third day and subsequent odd days; the key features in relation to even days is that feedback on behavior is provided, and the walking goal is reviewed. As illustrated in Figure 5, the step counts collected in “review tasks” is compared with the behavior goal, through verbal and visual feedback (2.2), *via* Vitoria’s speech and a chart (“assess” step). If the agreed goal has not been reached, Vitoria addresses barriers and proposes strategies to overcome them in the “counseling” step (problem-solving 1.2), as already explained in example C (Table 2). In Figure 6, Vitoria is portrayed implementing problem-solving (1.2). For instance, if the user chooses time constraints as a barrier, Vitoria recommends simple ways to improve step counts by integrating walking in the user’s routine, such as parking the car further away from the destination, selecting different routes to walk longer distances or using the stairs.

If the agreed goal is achieved, the intervention turns to the three basic psychological needs (competence, autonomy, and relatedness) in “counseling,” according to the ranking set up on day 2 for these constructs, as already explained. BCTs operationalized in this step are detailed in Table 2 (see example B).

**Figure 7:**
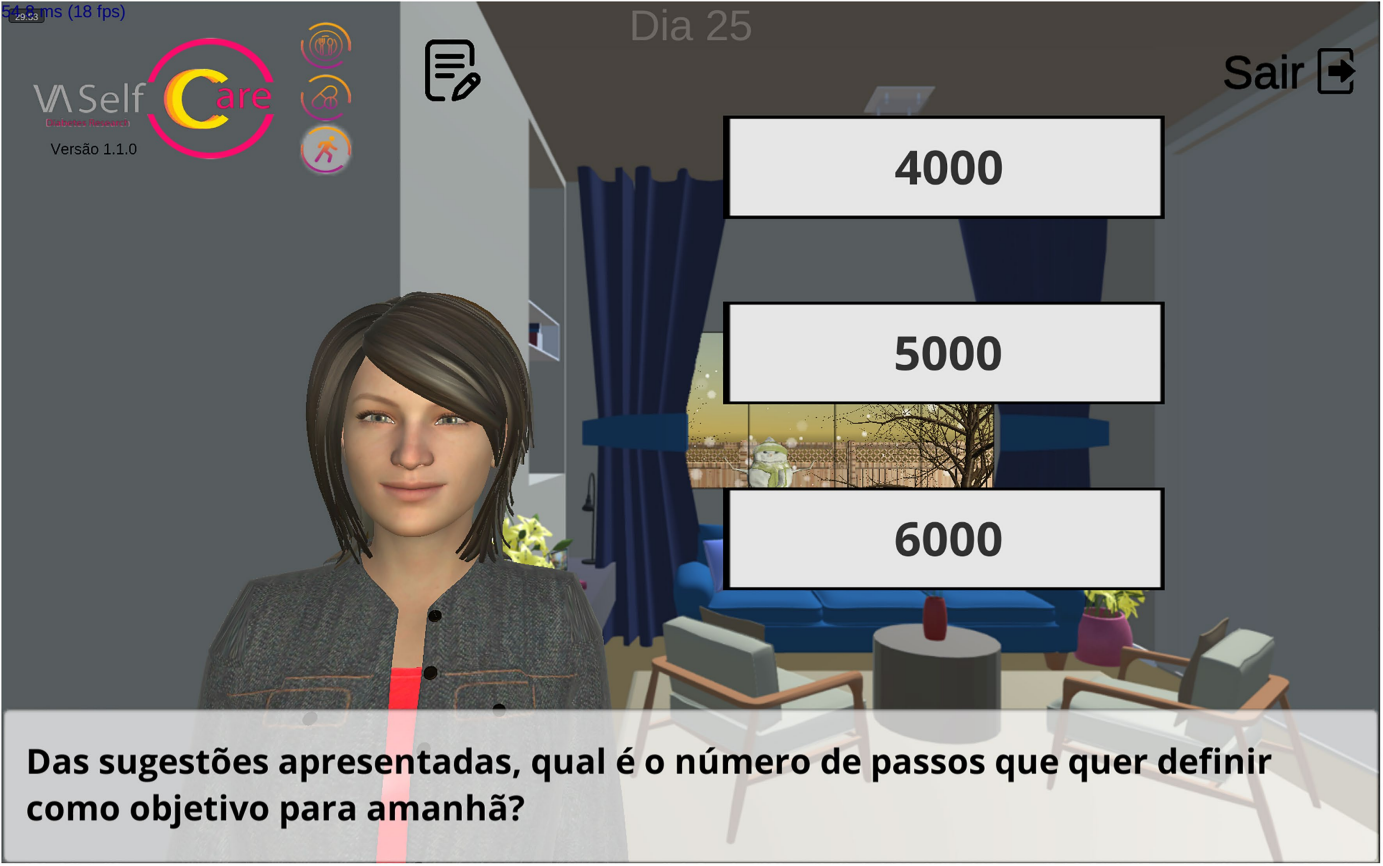
Interface in the mobile application: Vitoria reviewing the number of steps goal (review behavior goal 1.5). Translation to English: Of the suggestions presented, what is the number of steps you want to set as a goal for tomorrow? (subtitle).

Next, in the “assign tasks” the variation of step counts in relation to the agreed goal determines two paths (example D, Table 2). For the sake of illustration, if a user reports a count of 14,000 steps and the agreed goal was 6,000 steps, the variation is greater than 2000 (Δ ≥ ± 2000 steps). Vitoria asks whether the user wants to review the goal using the reported step counts or the agreed goal as the basis. Assuming the user selects the latter, the goal is reviewed using 6,000 steps as the basis, with increments above and below determined by the competence score (e.g., 5,500, 6,000, 6,500 steps). Figure 7 depicts another example.

#### “Lite” Physical Activity Component

The “lite” physical activity component, depicted in Figure 2, starts on the first day of the healthy eating component, when both the medication taking, and physical activity have been addressed more thoroughly. In essence, in the “lite” component, Vitoria collects information on step counts in each daily interaction but only assesses and counsels every 8 days; moreover, “assign tasks” set goals for the next week, and not for the next day.

**Figure 8:**
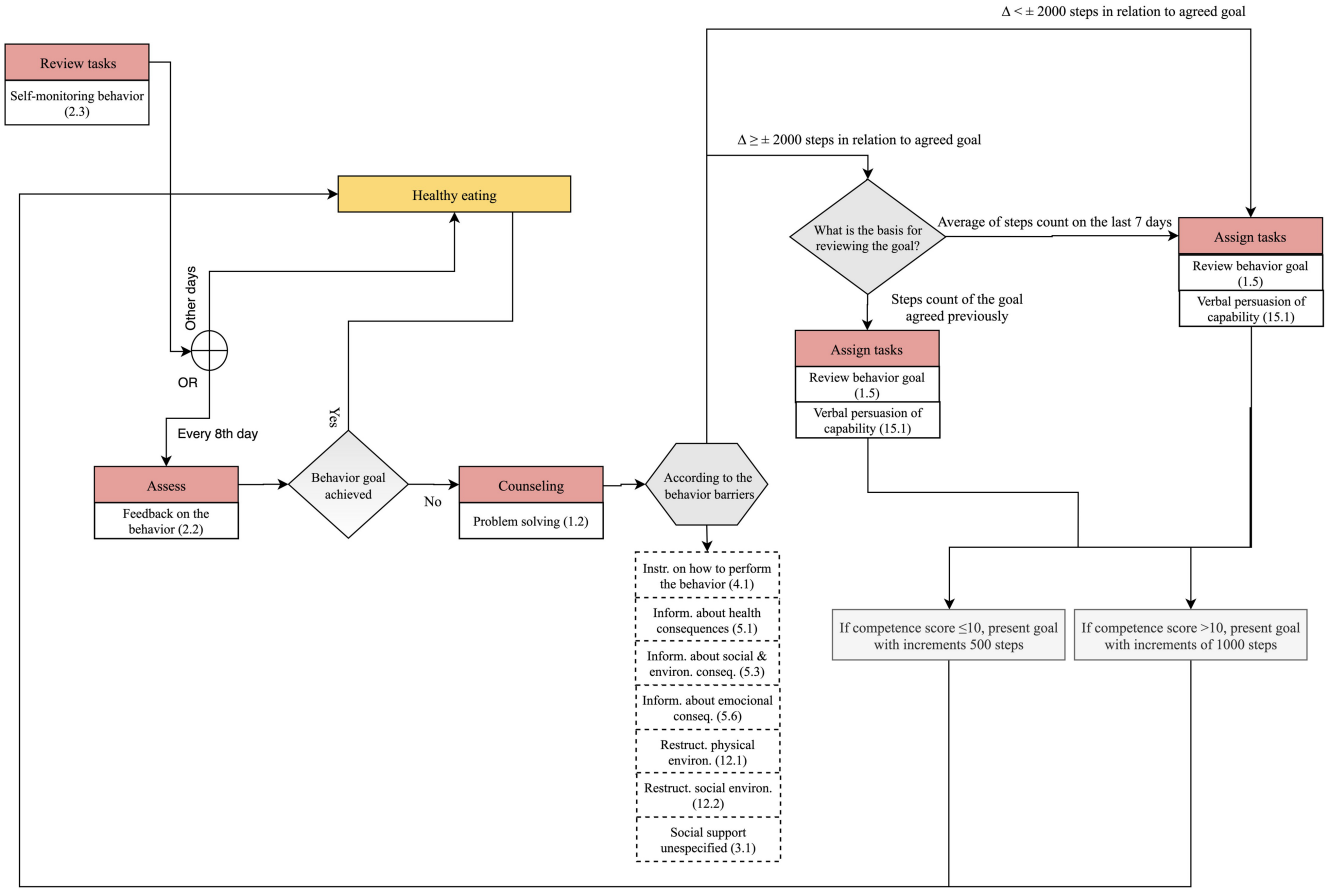
Dialogue flow in “lite” physical activity component.

Figure 8 presents only the dialogue steps regarding the “lite” version of the physical activity component. Vitoria starts by asking the user to input the step counts (“review tasks”). Then, the dialogue guides the user to the healthy eating component. This flow is repeated for 7 days. On the eighth day, Vitoria gives feedback on the average step counts of the last 7 days, which corresponds to the “assess” step (feedback on behavior 2.2).

Next, in the “assign tasks” the variation of step counts in relation to the average weekly goal determines two paths (example F, Table 2). For the sake of illustration, if a user has an average weekly count of 6,600 steps and the agreed goal for the week was 6,000 steps, the variation is smaller than 2000 (Δ < ± 2000 steps), the goal is reviewed using 6,600 steps as the basis, with increments above and below determined by the competence score (e.g., 5,600, 6,600, 7,600 steps).

## Discussion

This article illustrates an evidence and theory-based approach to specify the BCTs and their tailoring of an intervention to improve physical activity in older adults with T2D, subsumed in a multi-behavior intervention, *via* a mobile application with an anthropomorphic conversational agent.

While the design of the physical activity component drew on evidence and theory, there was a strong concern to keep a good fit with daily routine, by resorting to step counts as a measure of physical activity and by drawing on the multidisciplinary experience of the team to list facilitators and barriers.

Thirteen standardized BCTs from the BCTTv1 were chosen, based on meta-research and their practicality to the intervention. These BCTs were tailored in predefined steps of each interaction, using six if-then rules, which determine how the anthropomorphic conversational agent, Vitoria, interacts with users.

A limitation of our work is that users have to choose from a limited set of options when talking to Vitoria; moreover, BCTs are embedded in the dialogues in a rigid way, which limits the possibilities of tailoring. More recently, we attempted to overcome these issues by using an advanced natural language platform (e.g., Google Dialogflow) and an ontology-based knowledge representation, indicating how BCTs can be operationalized (Bastos et al., 2022).

Our work can also be criticized by the fact that the effectiveness of the digital intervention has not yet been evaluated. Usability testing with older adults living with T2D showed encouraging results (Balsa et al., 2020), but ultimately the merit of the intervention will be judged based on its ability to produce positive health outcomes. While a clinically significant decrease in glycated hemoglobin is typically warranted, humanistic health outcomes should not be demeaned. A paper provocatively entitled “If it does not significantly change HbA1c levels why should we waste time on it?,” reminds us of the perils of providing care to persons with diabetes contingent only upon achieving clinical outcomes (Jones et al., 2015). The only example that we are aware of a virtual human coach intervention for self-managing chronic disease resulted in statistically significant improvement in health-related quality of life, but not in glycated hemoglobin (Gong et al., 2020). This should be regarded as equally beneficial as improvements in glycated hemoglobin. The mean age of participants in the intervention group (*n* = 93) of this Australian randomized effectiveness-implementation trial was 55.4 years (SD 9.7; Gong et al., 2020), which reinforces the need for trialing our intervention in older adults, in a different context, leading to the accumulation of evidence on fully automated virtual human coaches. To determine trends in outcomes of interest, a protocol was drafted for a non-randomized non-controlled 3-month feasibility trial in nursing consultations in primary care. Endpoints, selected according to the International Consortium for Health Outcomes Measurement (ICHOM, 2019), included steps count as part of lifestyle factors, glycated hemoglobin for diabetes control, and psychological well-being as a patient-reported outcome. Self-monitoring of step counts resorting to pedometers is, on its own, beneficial to promote physical activity and related health outcomes (Bravata et al., 2007; Idowu et al., 2021). However, the need to input step counts manually in the mobile application prototype may be burdensome over time and can impact on intervention engagement and intervention fatigue, two important mechanisms influencing the use of mobile applications and retention (Nahum-Shani et al., 2018).

Just-in-time adaptive interventions (JITAIs) provide the right type (or amount) of support, at the right time, while eliminating support that is not beneficial, through continuous monitoring of the person’s state and context (Nahum-Shani et al., 2018). In what concerns physical activity, JITAIs are exemplified by prompting users for exercise at a particular time of the day if a certain accumulated steps count collected *via* passive assessment (e.g., *via* a smartphone accelerometer) has not been reached (Nahum-Shani et al., 2018). While self-report of step counts may undermine engagement over time and contribute to intervention fatigue, it has the advantage of improving the scalability of the intervention, by keeping it functional on a range of devices and simple to use. The latter is also important when attempting not to aggravate health inequalities. It has been suggested that the digital divide is shifting from access and connectivity to a knowledge gap on how to use information and communication technology (McAuley, 2014). This is particularly important for older adults, who are our target group. While global data show a consistent upward trend in smartphone penetration in those 65+, this does not necessarily translate in the use of mobile applications (Berenguer et al., 2017). Therefore, currently it appears sensible to keep applications for older adults as simple as possible. Usage data from trials in conjunction with qualitative explorations will shed light on older adults’ engagement with the VASelfCare digital intervention and their preferences.

An analysis of 16 mobile applications marketed for the prevention and management of T2D pleaded for more transparency in reporting the app features and employed BCTs (Keller et al., 2022). We believe the same plea should be made to researchers. We found little guidance from the scientific literature on granular aspects of intervention design and the operationalization of BCTs for digital behavior change interventions in T2D. For instance, the work of Gong et al. (2020), while providing much needed evidence on the effectiveness and implementation of a virtual human coach to support T2D self-management, offers little detail on the BCTs employed, hindering not only replication but also improvements in the intervention design and content.

The way in which the behavior change techniques are delivered is considered an element of behavior change interventions that warrants consideration as it can explain the (in-)efficacy of a given technique (Marques et al., 2020). Vitoria’s dialogues were created resorting to a helpful-cooperative communication style (Niess and Diefenbach, 2016), aiming to build rapport and trust. This non-judgmental approach is considered to increase autonomy and relatedness which are core basic psychological needs according to the Self-Determination Theory. Further, this communication style is in line with recommendations on the use of language to communicate with and about persons with diabetes, grouped under the umbrella of the Language Matters Diabetes global movement.^2^ These recommendations were developed based on evidence and expert opinion in countries such as Australia, United States and the United Kingdom (Dickinson et al., 2017; Cooper et al., 2018; Speight et al., 2021) and adapted for other countries, for guiding health professionals and other stakeholders (Batata et al., 2022). We believe there is room for applying the preferred language and principles entailed in these recommendations to digital behavior change interventions in T2D, to harness higher rapport and trust, aiming at higher engagement and effectiveness.

A final point meriting discussion is whether the VASelfCare prototype is appropriate to other cultures. Walking is a commonly accepted form of physical activity for both men and women in western societies, but maybe less common in other cultures. For example, research in India suggested that women associate physical activity mostly with household chores and do not contemplate walking as an exercise option, albeit finding it feasible (Mathews et al., 2016). This raises the point of cultural adaptation of digital behavior change interventions.

## Conclusion

Evidence and theory have been translated into an m-health prototype using an anthropomorphic conversational agent to promote physical activity in older adults with type 2 diabetes, as part of a multi-behavioral intervention. This approach, which includes 13 BCTs and six if-then rules that determine their tailoring and dialogue flow, is expected to maximize effectiveness and to facilitate replication. Ultimately, the present work may leverage the efforts of others in developing self-management interventions targeting lifestyle behaviors.

## Data Availability Statement

The original contributions presented in the study are included in the article/supplementary material, further inquiries can be directed to the corresponding author.

## Author Contributions

IF, NP, and MG conceived the work reported in this paper. IF and NP led the design of the physical activity component, supported by MG, DM, and MM. IF, NP, and MG wrote the first draft of the manuscript. MM performed a first critical review. Subsequent iterations of the manuscript were commented on by all authors. All authors contributed to the article and approved the submitted version.

## Funding

This project was supported by FCT and Compete 2020 (grant number LISBOA-01-0145-FEDER-024250, 02/SAICT/2016).

## Conflict of Interest

The authors declare that the research was conducted in the absence of any commercial or financial relationships that could be construed as a potential conflict of interest.

## Publisher’s Note

All claims expressed in this article are solely those of the authors and do not necessarily represent those of their affiliated organizations, or those of the publisher, the editors and the reviewers. Any product that may be evaluated in this article, or claim that may be made by its manufacturer, is not guaranteed or endorsed by the publisher.

## Footnotes

^1^ https://vaselfcare.esel.pt

^2^ https://www.languagemattersdiabetes.com
